# Meachem on Dysentery

**Published:** 1849-02

**Authors:** T. G. Meachem


"*Meachem on Dysentery.*"—Under this caption there appeared in the Editorial department of the last number of this Journal, an article from the pen of "W T.," designed, with the exception of an equivocal compliment, or two, which it pays to the Ontario Medical Society and myself, to expose "the inadvertence so frequently met with," into which those physicians had fallen—to whose practice reference is made in my Address published in a preceding No. of the Journal: "Some relied mainly on astringents, such as acetate of lead combined with Dover's powder." The remarks of "W T." respecting this combination, are based on the supposition that Dover's powder is an union of ipecac, opium, and *sulphate of potassa.* This, it is true, is the officinal preparation known by that name, as directed by the Dispensatory of Wood and Bache—though differing somewhat from the prescription left by the inventor of the medicine, Dr. Dover—and were *it* to be combined with acetate of lead, an insoluble, and inert sulphate of lead would be formed, as "W. T." observed. But there is a preparation called Dover's powder, used extensively in this section, Vermont, and I doubt not, other portions of the country, which varies from the offinical compound in containing *nitrate* of Potassa as a substitute for *sulphate* of potassa, an article with which, I believe, the acetate of lead is not incompatible. This kind of Dover's powder it was, which was used, certainly by *many*, and probably by *most*, of the physicians whose practice "W. T." condemns; so that, instead of administeriting an incompatible compound, whose product is an insoluble sulphate of, lead, they prescribed, scientifically and wisely, a combination of congruous medicines, aptly adapted to the cases under their charge. T. G. MEACHEM.

